# Psychological Changes and Cancer Occurrence in Seoul Citizens Due to Changes in Fine Dust Concentration before Seoul Fine Dust Policy

**DOI:** 10.3390/ijerph182111210

**Published:** 2021-10-25

**Authors:** Kilyong Choi, Wonho Yang

**Affiliations:** 1 Department of Environmental Energy Engineering, Anyang University, Anyang 14028, Korea; bestchoi94@anyang.ac.kr; 2 Department of Occupational Health, Daegu Catholic University, Daegu 38430, Korea

**Keywords:** environmental diseases, air pollution, fine dust, living environment, satisfaction, policy

## Abstract

Background: Particulate matter and urban air pollution affect the human body and can lead to death. Epidemiological studies should consider exposure to pollutants and the diverse responses of individuals, depending on their sensitivity to the pollutants. Methods: In this study, air pollution measurements were obtained hourly at measuring stations operated by national and local governments to increase the reliability of the measured values. A β-ray absorption method was used to analyze the measurements of fine dust and determine the particulate matter content. Results: The air pollution data were log-linear, thereby enabling a comparison of data from different time periods. The comparison was made by focusing on the period of the implementation of Seoul’s fine dust policy. It was observed that the cancer incidence rate decreased after the implementation of the policy. The data on individual characteristics were obtained from a survey of Seoul citizens conducted from 2015 to 2016 using indicators such as quality of life and the social trust of Seoul citizens. Conclusion: The survey on the living environment and residence indicated that 80% of the heads of households were men. Women had a greater dissatisfaction than men with their residential, economic, and social environments. The survey questions on well-being were related to elements of internal and external environments, such as air pollution, noise, and fine dust.

## 1. Introduction

In recent years, a number of studies have been published on various respiratory symptoms caused by air pollution and the associated psychological and physiological evaluation of affected individuals [1,2]. The human body is greatly influenced by the environment, and various studies have been conducted to determine methods with which to prevent exposure to various environmental elements that are present in the atmosphere as a result of environmental changes. Particulate matter (PM) and urban air pollution affect the human body and can lead to death [3,4,5,6]. The concentration of PM (coarse PM (PM10) and fine PM (PM2.5)) in the atmosphere has been investigated in relation to respiratory and cardiovascular diseases, and correlations between these diseases and the presence of fine dust have been reported [7,8]. Epidemiological studies on air pollution should consider the exposure to pollutants and the diverse responses of individuals, depending on their sensitivity to the pollutants. Air pollution is very sensitive to temperature and meteorological changes, and, as such, studies should take into account the changes in these factors and their impact on results [9]. Seasonal changes in relation to air pollution, regional characteristics, and population distribution are considered important variables not only in Korea but also worldwide [10]. Climate change has a direct effect on the length and timing of the seasons. The prevention of the adverse health effects of environmental pollutants is required to address problems such as diseases and mortality [11]. Studies have provided evidence that the fear of exposure to various air pollutants can cause psychological effects; these studies focused on the analysis of air pollution factors affecting seasonal disease occurrence and the treatment of such diseases [12,13]. In addition, health concerns about air pollution are changing people’s attitudes toward outdoor activities, causing confusion, increasing distrust in society and the state, and causing ideological conflict.

For the purpose of research, the Seoul Metropolitan Government has been implementing a policy process for a fine dust season system since 2015, and the aim of this study is to check impact. There are some data on atmospheric exposure and health indicators, but there has been no study confirming the correlation between recent abnormal symptoms and health anxiety factors. This study is based on the hypothesis that the correlation between PM10 and PM2.5 concentrations in Seoul, Gyeonggi, and Incheon varies seasonally. The data indicate that the population in the residential areas in Seoul is directly affected by air pollution, noise, lack of greenery, and water pollution.

## 2. Materials and Methods

### 2.1. Survey

A survey of Seoul citizens was conducted from 2015 to 2016 using 227 indicators (12 areas and 42 items) of quality of life, social trust, and community consciousness. The survey included questions regarding satisfaction with the living environment. According to the 2016 data, the score for satisfaction with the residential environment (e.g., water supply and sewage, housing, telecommunication, traffic, green areas) was the highest (6.16 points out of 10 points), satisfaction with the social environment (e.g., welfare, disease, medical facilities) scored 5.71 points, satisfaction with the educational environment scored 5.43 points, and satisfaction with the economic environment scored 5.31 points. Satisfaction based on gender was determined. The highest score was observed for the residential environment and was influenced by the factors of waterworks, sewage, housing, electricity, communication, traffic, and greenery. In the category of daily life concerns, problems related to parking had the highest proportion (49.1%), followed by street litter (40.4%), crime and violence (32.8%), and air pollution (32.3%). The low values for air pollution and water quality issues are important when considering the immediate and future impacts of fine dust.

### 2.2. Air Pollution Data

Airborne pollution data were obtained hourly from national and local government measuring stations. The average annual concentration (less than 50 μg/m^3^) and the average daily concentration (less than 100 μg/m^3^) of fine dust (PM10 and PM2.5) were measured using the β-ray absorption method. This method captures PM of 10 μm or less in the air (the particle size can be controlled according to the separation device). The sample is placed on filter paper for a certain period of time and permeates the betaine. The weight concentration of the PM is measured continuously. This measurement method determines the β-radiation absorbed by the dust particles on the filter paper. The analysis was conducted based on the following formula:

I = Io ing foμ X(1)

I: β-ray intensity transmitted through the dust on the filter paper;

Io: β-ray intensity transmitted through a blank filter paper;

μ: absorption coefficient of β-ray absorption by dust (cm^3^/mg);

X: mass of the collected basin per unit area (mg/cm^3^).

Therefore, dust concentration was determined by the amount of absorption of beta rays by the mass of dust collected per unit area. Dust concentration was determined by:

C = (S / μ×V×△t) In(I/Io)(2)

C: dust concentration (mg/ m^3^);

S: area of filter paper (cm^2^);

V: amount of air absorbed (m^3^);

△t: collection time (min).

### 2.3. Statistics

Data analysis was performed using the statistical software SAS version 9.4 (SAS Institute Inc., Cary, NC, USA). We analyzed the parameters for life satisfaction (living environment, economic environment, and social environment) and levels of well-being (noise, air pollution, rest spaces, lack of greenery, and water pollution). Gender differences were analyzed. A logistic regression model was used to compare the seasonal changes and regional characteristics of the PM10 and PM2.5 concentrations near the homes of the subjects. A 95% confidence interval (CI) and probability ratios (odds ratios (ORs)) were used to determine significant differences. A *p*-value <0.05 was considered statistically significant. The dependent variables were the individual characteristics and local dust (PM10 and PM2.5) concentrations. The independent variables were divided into categories representing gender and changes in summer.

### 2.4. Diagnostic Code Analysis of Cancer Registration Data

The sample of this study is a cohort of residents of Seoul, Incheon, and Gyeonggi-do, and, as such, it is difficult to identify and epidemiologically approach the short-term impacts. With regard to policy application and management, due to the characteristics of Seoul, the purpose of the protocol, according to the observation of accumulation of dust concentrations as the cause of disease determined in terms of outcome, was defined as follows: The customized DB confirmed the health insurance claim data (qualification and treatment DB) of all persons with a residence code from 2009 to 2019. The cohort type was an open cohort according to residential area by year after the start of follow-up (2009). This study analyzed 15 cancers (all cancers (C00–C96), lung cancer (C33–C34), stomach cancer (C16), liver cancer (C22), colorectal cancer (C18–C20), breast cancer (C50), cervical cancer (C53), cancer of the esophagus (C15), gallbladder cancer (C23–C241), pancreatic cancer (C25), laryngeal cancer (C32), small intestine cancer (C17), skin cancer (C44), renal cancer (C64–C68), leukemia (C91–C95), and thyroid cancer (C73)) that were found to be induced by the environment among the representative cancers (24 types) in Korea.

## 3. Results

### 3.1. Living Environment and Residence

The results shown in Table 1 are related to the living environment of the survey subjects. First, in the survey of heads of households, males accounted for about 80% of survey subjects during the period of 2015–2016, a much higher proportion than females. According to the income characteristics of Seoul, approximately 30% of participants with an education of 15 years earned an average of KRW 40 million. During the study period, the number of households with an income above KRW 5 million was higher than in other years, and the income of participants with 15 or 16 years of education was notably higher than that those with a lower level of education. Thus, an education of more than 16 years was not investigated further. We could confirm that the survey respondents in Seoul have a high level of education and a high income.

Table 2 displays satisfaction with the living environment (living environment, economic environment, and social environment) and well-being (e.g., noise, air pollution, resting space, water pollution). The survey data for 2015 to 2016 indicated satisfaction with various factors related to the living environment. Life safety was significant in the data for 2015 data.

### 3.2. Outdoor Environment

The raw data from the outdoor air pollution monitoring network were compared with the log-linear data. The results presented in Table 3, Table 4 and Table 5 are based on the analysis of fine dust, which is one of the variables affecting the living environment. In 2015, the PM10 concentrations for Gyeonggi and Incheon were 3.73 μg/m^3^, and the PM2.5 concentration was 3.24 μg/m^3^ in Incheon and 3.04 μg/m^3^ in Gyeonggi; the PM2.5 concentration was low in Seoul (2.91 μg/m^3^).

**Figure 1:**
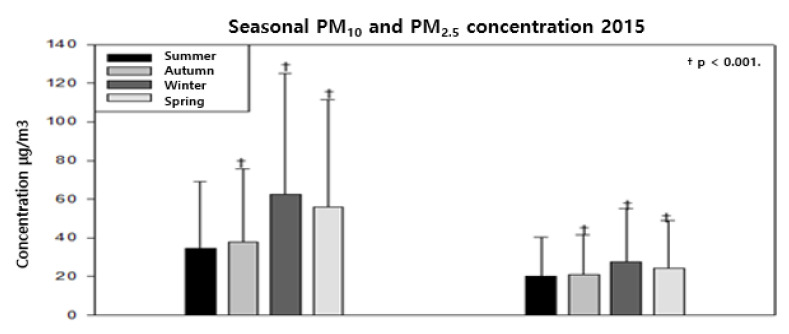
Correlation between the concentration (log-linear) of seasonal fine dust (PM10 and PM2.5) and the Seoul area (2015).

**Figure 2:**
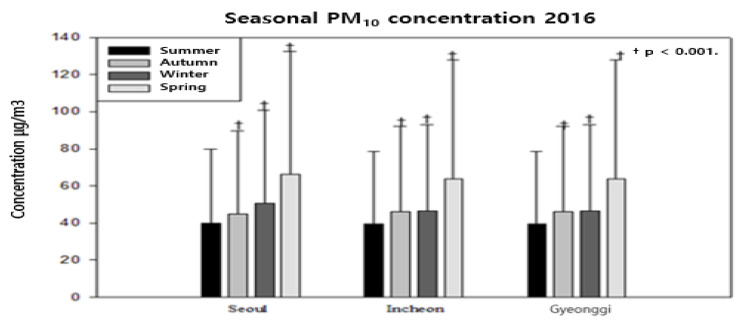
Correlation between the concentration (log-linear) of seasonal fine dust (PM10) and the Seoul area (2016).

**Figure 3:**
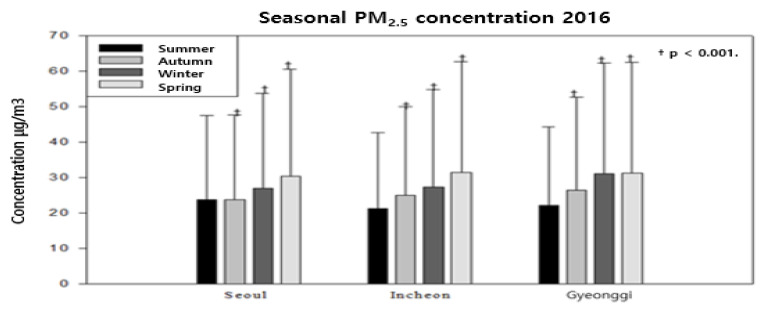
Correlation between the concentration (log-linear) of seasonal fine dust (PM2.5) and the Seoul area (2016).

As shown in Table 4 and Table 5, the concentration of fine dust in the Seoul area tends to be higher in the autumn, winter, and spring than in the summer. This is consistent with the results of other studies. The ORs of the seasonal average PM10 concentration in 2016 are 1.47 in autumn, 2.11 in winter, and 4.59 in spring. In Incheon, the ORs are 1.65 in the autumn, 1.60 in the winter, and 4.21 in the spring. The Gyeonggi area showed a trend of increasing seasonal fine dust concentrations (2.26 in the autumn, 3.45 in the spring, and 6.16 in the winter). The values were statistically significant (*p*-value <0.0001) for the autumn of 2016. The OR for PM2.5 was 0.98 in the autumn (statistically insignificant) and increased to 1.19 in the winter and 1.66 in the spring (1.50, 1.64, and 2.48 in Incheon, respectively). The OR values for PM10 for Gyeonggi were 1.49, 2.27, and 2.32, respectively, and were lower than the values for PM10 but were statistically significant (*p*-value <0.0001). In 2015, the OR values for PM10 in the autumn, winter, and spring in Seoul were 1.36, 5.77, and 6.04, respectively. In Incheon, the autumn, winter, and spring values were 1.19, 2.74, and 3.41, respectively. In Gyeonggi, the autumn, winter, and spring values were 1.65, 4.13, and 3.23, respectively (Figure 1, Figure 2 and Figure 3). As shown in Figure 1, PM10 in Seoul showed a continuous increase to 1.36 (1.26–1.47), 5.77 (5.36–6.2), and 6.04 (5.60–6.51) in Autumn, Winter, and Spring, respectively, compared to Summer. And in PM2.5 of Seoul, there was a statistically significant high trend in Winter and Spring with 3.02 (2.78–3.28) and 1.72 (1.58–1.88), respectively. As shown in Figure 2, the result of checking PM10 in Seoul. Compared to Summer, it was confirmed that the risk increased to 1.47 (1.44–1.50), 2.11 (2.07–2.16), and 4.59 (4.49–4.68) in Autumn, Winter, and Spring, respectively, which was statistically significant.

As shown in Figure 3. The result of checking PM2.5 in Seoul. It was confirmed that the risk increased to 1.19 (1.16–1.22) and 1.66 (1.62–1.70), respectively, in Winter and Spring compared to Summer, which was statistically significant.

**Figure 4:**
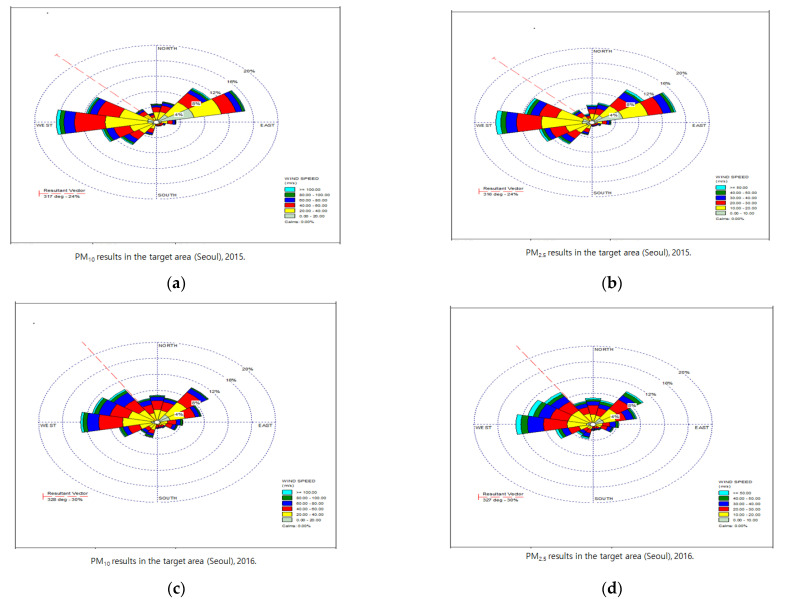
Correlation between the concentration (log-linear) of fine dust (PM10 and PM2.5) and the local climate (wind). (**a**) PM10 results in the target area (Seoul), 2015. (**b**) PM2.5 results in the target area (Seoul), 2015. (**c**) PM10 results in the target area (Seoul), 2016. (**d**) PM2.5 results in the target area (Seoul), 2016.

As a result of the environmental data centered on the above data, the effect of fine dust on the characteristics of climate change and regions (Seoul, Gyeonggi, and Incheon) had the same tendency. Moreover, the influence of wind confirmed the direct characteristics of fine dust. Winds were blowing north and west, which had a direct effect, confirming that the impact on China was influenced by Seoul and Gyeonggi Province. These results confirm the new validation of previous studies and can be used as reliable data (Figure 4).

As shown in Table 6 in Seoul, there are many external factors for fine dust caused by Korean vehicles and external small business establishments. Accordingly, it was confirmed that the incidence of cancer was higher than that in Gyeonggi, which has more industrial complexes than Seoul, and Incheon, which has a high impact of fine dust and chemicals from ports. There was a statistical effect on all cancers, but it was not statistically significant in cervical cancer. As a result of confirming the cancer incidence rate for the seasonal policy of fine dust in urban areas, about 3% of all cancers showed a continuous increase. In the first year (2019) of applying the fine dust policy, it was confirmed that the cancer incidence rate increased by 1%. Moreover, after the implementation of the policy, fine dust decreased for lung cancer (C33–C34), breast cancer (C50), laryngeal cancer (C32), small intestine cancer (C17), skin cancer (C44), and thyroid cancer (C73) (after policy implementation: 2.7%, 1.8%, 2.2%, 1.6%, 1.2%, and 1.8%, respectively; before policy implementation: 3.0%, 1.9%, 2.8%, 1.8%, 1.7%, 1.9%, respectively). In addition, there was no significant difference in fine dust concentration between the start of the fine dust policy (2015) and the period during which it was applied (2019), although the concentration of fine dust was notably high in 2018.

## 4. Discussion

Various results and indicators in a study of fine dust pollution from 2011 to 2015 were similar to the results of our study based on the economic indicators of the Seoul metropolitan area [14]. Other studies have found that monthly changes in the concentration of fine dust are related to the seasons; the concentrations increased in November, peaked in February, and then decreased gradually and reached their lowest levels in August and September [15,16]. This is due to the effect of rain, wind, and weather changes during the summer, resulting in low dust concentrations. In the winter, the use of indoor and outdoor fuel increases due to heating. The polluted air does not circulate due to air congestion, and the influence of air pollutants on the living environment thereby increases. Therefore, the concentration of fine dust is higher in the winter [17]. Accordingly, since 2015, the Seoul Metropolitan Government has regulated the fine dust management policy through discussions. Since then, fine dust has gradually decreased, but there has been no change in environmental diseases. Among such diseases, according to cancer-related information, an increasing number of cancer cases has been observed. There are three major findings of this study. In our hypothesis, we considered various demographic, geographical, and socioenvironmental factors unique to Korea, including high excessive population density, the geographical characteristics of neighboring countries, and the presence of industrial parks in Incheon and Gyeonggi. There are a few areas that are not affected by fine dust. In addition, it has been predicted that the risk of fine dust pollution in Seoul will increase in the coming years [18,19]. Second, it was confirmed that the fine dust concentration and PM content exhibited seasonal fluctuations; the PM content was lower in the summer and autumn when the precipitation was higher. These results should be taken into consideration in research studies and policy development in the future [20]. It could be confirmed that future management is necessary when examining the decrease in environmental diseases due to policy changes, such as lung cancer and skin cancer. Finally, unlike other areas, Seoul is a densely populated area, so it will have to make an effort to achieve balanced regional development in the future. In 2019, the first fine dust policy was only applied to the operation of vehicles and large buildings. In the future, it will be necessary to examine various variables that contribute to population movement and balanced regional development. Therefore, it will be necessary to determine the effect of air pollution on the perception of the public due to the psychological impact on the living environment and the welfare of the residents in Seoul, and it will be necessary to evaluate the significant difference in the reduction in cancer incidence.

## 5. Conclusions

The aims of this study were to determine whether seasonal changes in relation to air pollutants according to Seoul’s air policy are well managed and the extent to which they affect the health of Seoul citizens. The results of this study cannot confirm the characteristics and prevalence of all regions according to seasonal characteristics. Although there is no direct relationship between the time of the questionnaire and the results of the measurements of fine dust concentrations, it is suggested that this relationship should be taken into account in future studies considering the psychological aspects and their specificity. The statistical analysis showed that the seasonal characteristics of fine dust were significant. Thus, the results may be used as important data to confirm various changes, depending on the extent to which changes in seasons reflect Seoul’s air policy, and to determine whether an extension of the policy period in the future should be considered. Previous studies have indicated that the causes of the lower concentrations of fine dust in summer were rainfall and weather conditions (wind). These fluctuations were also observed in the neighboring areas of the Seoul metropolitan area (Gyeonggi and Incheon). Satisfaction with the living environment was very low for women with regard to the residential environment, economic environment, and social environment. After the implementation of the policy, the incidence of cancer showed a decreasing trend for lung cancer and other cancers in some environments. In the future, it will be necessary to determine the correlation between fine dust and health and cancer, with consideration of psychological factors. The accuracy of these results could be improved if accurate real-time measurements of dust concentrations and individual risk information from exposure to air pollutants were available. In addition, measures for disease management should be improved.

## Figures and Tables

**Table 1:** Gender, income, and education levels of surveys respondents in Seoul.

Gender	Questionnaire for 2016 (Head of Household)		Questionnaire for 2015 (Head of Household)		Questionnaire for 2014 (Head of Household)	
		*p*-Value		*p*-Value		*p*-Value
Men	16,626 (83.1)		17,326 (86.6)		16,931 (84.7)	
Women	3374 (16.9)		2674 (13.4)		3069 (15.3)	
Income				<0.0001		<0.0001
200>	1876 (9.4)		1273 (6.4)		2471 (12.4)	
201–400	6520 (32.6)		4768 (23.8)		7792 (39.0)	
401–500	7322 (36.6)		3418 (17.1)		4532 (22.6)	
501<	4282 (21.4)		10,541 (52.7)		5205 (26.0)	
Education	-			<0.0001		<0.0001
Middle school graduation	-		1353 (6.8)		2012 (10.1)	
High school graduation	-		7323 (36.6)		6551 (32.7)	
University graduation	-		10,983 (54.9)		11,096 (55.5)	
Graduate school graduation	-		341 (1.7)		341 (1.7)	

**Table 2:** Living environment and well-being in Seoul.

Life Environment Satisfaction	Questionnaire for 2016 (Households by Gender)		Questionnaire for 2015 (Households by Gender)		Questionnaire for 2014 (Households by Gender)	
	Crude OR (95% CI) *	*p*-Value	Crude OR (95% CI) *	*p*-Value	Crude OR (95% CI) *	*p*-Value
Residential environment **	3.41 (3.38, 3.44)	<0.0001 †	3.43 (3.41, 3.46)	0.0164 ‡	3.40 (3.38, 3.43)	<0.0001 †
Economic environment ***	3.03 (3.01, 3.06)	<0.0001 †	3.02 (2.99, 3.05)	<0.0001 †	3.02 (2.99, 3.05)	<0.0001 †
Social environment ****	3.245 (3.22, 3.27)	<0.0001 †	3.24 (3.21, 3.27)	0.0034 ‡	3.23 (3.20, 3.26)	<0.0001 †
Noise	2.16 (2.14, 2.19)	0.3872	2.18 (2.15, 2.20)	0.6399	6.42 (6.30, 6.55)	<0.0001 †
Air pollution	2.20 (2.17, 2.22)	0.8011	2.17 (2.14, 2.20)	0.0226 ‡	3.72 (3.53, 3.90)	0.0397 ‡
Relaxation space	2.12 (2.10, 2.14)	0.9974	2.14 (2.11, 2.16)	0.1891	4.70 (4.47, 4.93)	0.3714
Water pollution	2.07 (2.04, 2.09)	0.3105	2.06 (2.03, 2.08)	0.1131	5.42 (5.16, 5.68)	0.3106

* Women’s crude OR (95% CI: confidence interval). ** Water and sewage, housing, electrical, communication, traffic, and green space. *** Living expenses, income, and working hours. **** Welfare, disease, and medical facilities. ‡ *p* < 0.05; † *p* < 0.001.

**Table 3:** Concentration (log-linear) of fine dust (PM10 and PM2.5) in Seoul, Incheon, and Gyeonggi.

Area and Measurement Target	Fine Dust Concentration in 2016 (μg/m^3^)				Fine Dust Concentration in 2015 (μg/m^3^)				Fine Dust Concentration in 2014 (μg/m^3^)			
	*n*	Minimum	Maximum	Average	*n*	Minimum	Maximum	Average	*n*	Minimum	Maximum	Average
Seoul PM10	332,331	0	6.29	3.76	27,046	0	6.94	3.63	339,171	0	5.7	3.67
Seoul PM2.5	212,070	0	5.1	3.08	18,195	0	5	2.91	-	-	-	-
Incheon PM10	176,097	0	6.87	3.73	24,328	0	6.86	3.73	172,484	0	5.75	3.74
Incheon PM2.5	119,060	0	5.41	3.02	7771	0	5.06	3.24	-	-	-	-
Gyeonggi PM10	686,650	0	6.6	3.8	24,549	1.1	6.73	3.73	676,932	0	6.99	3.8
Gyeonggi PM2.5	218,809	0	5.48	3.11	11,155	0	5.16	3.04	-	-	-	-

**Table 4:** Seasonal and average fine dust (PM10 and PM2.5) concentrations (log-linear).

Area and Measurement Target	Fine Dust Concentration in 2016 (μg/m^3^)				Fine Dust Concentration in 2015 (μg/m^3^)			Fine Dust Concentration in 2014 (μg/m^3^)		
	Season	Average Concentration		*p*-Value	Average Concentration		*p*-Value	Average Concentration		*p*-Value
		*n* = Low (%)	*n* = High (%)		*n* = Low (%)	*n* = High (%)		*n* = Low (%)	*n* = High (%)	
Seoul PM10	Summer	54,316 (33.1)	28,829 (17.1)	<0.0001 †	4826 (35.3)	1697 (12.7)	<0.0001 †	54,924 (32.7)	31,544 (18.4)	<0.0001 †
	Autumn	45,731 (27.9)	35,641 (21.2)		4174 (30.5)	1996 (14.9)		52,598 (31.3)	31,561 (18.4)	
	Winter	39,321 (24)	44,104 (26.2)		2571 (18.8)	5212 (39.0)		35,719 (21.3)	46,474 (27.1)	
	Spring	24,574 (15)	59,815 (35.5)		2104 (15.4)	4466 (33.4)		24,604 (14.7)	61,747 (36.0)	
Seoul PM2.5	Summer	28,731 (27.2)	24,476 (23)	<0.0001 †	2583 (27.8)	1612 (18.1)	<0.0001 †	ND		
	Autumn	28,086 (26.6)	23,326 (21.9)		2714 (29.2)	1408 (15.8)				
	Winter	26,511 (25.1)	26,930 (25.3)		1935 (20.8)	3644 (41.0)				
	Spring	22,346 (21.1)	31,664 (29.8)		2072 (22.3)	2227 (25.1)				
Incheon PM10	Summer	28,290 (32.6)	16,071 (18.0)	<0.0001 †	3733 (30.4)	1835 (15.3)	<0.0001 †	26,236 (30.2)	15,839 (18.5)	<0.0001 †
	Autumn	22,994 (26.5)	21,535 (24.2)		2828 (23)	1657 (13.8)		26,039 (30.0)	18,803 (22.0)	
	Winter	22,641 (26.1)	20,555 (23.0)		3277 (26.6)	4416 (36.7)		20,766 (23.9)	21,367 (25.0)	
	Spring	12,985 (14.9)	31,026 (34.8)		2459 (20)	4123 (34.3)		13,900 (16.0)	29,534 (34.5)	
Incheon PM2.5	Summer	18,873 (32.1)	12,473 (20.7)	<0.0001 †	991 (25.2)	1034 (27.0)	<0.0001 †	ND		
	Autumn	17,289 (29.4)	17,155 (28.4)		1244 (31.6)	881(23.0)				
	Winter	11,635 (19.8)	12,644 (21.0)		1065 (27.0)	1064 (27.8)				
	Spring	10,997 (18.7)	17,994 (29.9)		640 (16.2)	852 (22.2)				
Gyeonggi PM10	Summer	124,278 (36.0)	46,156 (13.5)	<0.0001 †	3303 (26.6)	1332 (11.0)	<0.0001 †	107,465 (32.1)	63,758 (18.7)	<0.0001 †
	Autumn	91,757 (26.6)	76,913 (22.5)		3318 (26.7)	2209 (18.2)		104,968 (31.3)	62,470 (18.3)	
	Winter	75,875 (22)	97,315 (28.5)		2912 (23.5)	4848 (39.9)		72,127 (21.5)	92,844 (27.2)	
	Spring	53,044 (15.4)	121,312 (35.5)		2878 (23.2)	3749 (30.9)		50,451 (15.1)	122,849 (35.9)	
Gyeonggi PM2.5	Summer	34,513 (31.0)	20,402 (19)	<0.0001 †	1206 (22.1)	717 (12.6)	<0.0001 †	ND		
	Autumn	36,525 (32.8)	32,101 (29.9)		1722 (31.6)	1079 (18.9)				
	Winter	20,707 (18.6)	27,826 (25.9)		1532 (28.1)	2712 (47.5)				
	Spring	19,699 (17.7)	27,036 (25.2)		991 (18.2)	1196 (21.0)				

† *p* < 0.001.

**Table 5:** Correlation (OR) between the concentration of average fine dust (PM10 and PM2.5) and season (log-linear).

Area and Measurement Target	Fine Dust Concentration, 2016 (μg/m^3^)			Fine Dust Concentration, 2015 (μg/m^3^)		Fine Dust Concentration, 2014 (μg/m^3^)	
	Season	OR (95% CI) *	*p*-Value	OR (95% CI) *	*p*-Value	OR (95% CI) *	*p*-Value
Seoul PM10	Summer	1		1		1	
	Autumn	1.47 (1.44–1.50)	<0.0001 †	1.36 (1.26–1.47)	<0.0001 †	1.05 (1.02–1.07)	<0.0001 †
	Winter	2.11 (2.07–2.16)	<0.0001 †	5.77 (5.36–6.2)	<0.0001 †	2.27 (2.22–2.31)	<0.0001 †
	Spring	4.59 (4.49–4.68)	<0.0001 †	6.04 (5.60–6.51)	<0.0001 †	4.37 (4.28–4.46)	<0.0001 †
Seoul PM2.5	Summer	1		1		ND	
	Autumn	0.98 (0.95–1.00)	0.1389	0.83 (0.76–0.91)	<0.0001 †		
	Winter	1.19 (1.16–0.22)	<0.0001 †	3.02 (2.78–3.28)	<0.0001 †		
	Spring	1.66 (1.62–1.70)	<0.0001 †	1.72 (1.58–1.88)	<0.0001 †		
Incheon PM10	Summer	1		1		1	
	Autumn	1.65 (1.61–1.69)	<0.0001 †	1.19 (1.10–1.30)	<0.0001 †	1.20 (1.16–1.23)	<0.0001 †
	Winter	1.60 (1.56–1.64)	<0.0001 †	2.74 (2.55–2.95)	<0.0001 †	1.70 (1.66–1.75)	<0.0001 †
	Spring	4.21 (4.09–4.33)	<0.0001 †	3.41 (3.16–3.68)	<0.0001 †	3.52 (3.42–3.62)	<0.0001 †
Incheon PM2.5	Summer	1		1		ND	
	Autumn	1.50 (1.46–1.55)	<0.0001 †	0.68 (0.6–0.77)	<0.0001 †		
	Winter	1.64 (1.59–1.70)	<0.0001 †	0.96 (0.85–1.08)	0.9293		
	Spring	2.48 (2.40–2.56)	<0.0001 †	1.28 (1.12–1.46)	<0.0001 †		
Gyeonggi PM10	Summer	1		1		1	
	Autumn	2.26 (2.23–2.29)	<0.0001 †	1.65 (1.52–1.79)	<0.0001 †	1.00 (0.99–1.02)	<0.0001 †
	Winter	3.45 (3.40–3.50)	<0.0001 †	4.13 (3.82–4.47)	<0.0001 †	2.17 (2.14–2.20)	<0.0001 †
	Spring	6.16 (6.07–6.25)	<0.0001 †	3.23 (2.98–3.50)	<0.0001 †	4.10 (4.05–4.16)	<0.0001 †
Gyeonggi PM2.5	Summer	1		1		ND	
	Autumn	1.49 (1.45–1.52)	<0.0001 †	1.05 (0.94–1.19)	<0.0001 †		
	Winter	2.27 (2.22–2.33)	<0.0001 †	2.98 (2.66–3.33)	<0.0001 †		
	Spring	2.32 (2.26–2.38)	<0.0001 †	2.03 (1.79–2.3)	<0.0001 †		

* Odds ratio 95% confidence limits: † *p* < 0.001.

**Table 6:** Risk ration of major cancers according to the fine dust policy in the downtown area of Seoul.

Cancer Type (Code Number)	Year (Number)											*p*-Value
All Cancers (C00–C96)	2009	2010	2011	2012	2013	2014	2015	2016	2017	2018	2019	
Seoul	232,751	208,592	241,802	276,896	305,612	330,957	352,633	375,132	400,161	424,802	452,533	-
Incheon	33,795	44,527	51,986	62,045	70,258	77,642	85,519	93,990	102,506	111,234	120,087	0.0007 †
Gyeonggi	159,981	210,371	243,225	286,422	321,999	355,916	390,624	433,498	475,164	520,006	567,114	0.0008 †
Lung cancer (C33–C34)												
Seoul	7292	5615	6635	8415	9393	10,356	11,300	12,334	14,195	16,028	17,872	-
Incheon	1015	1174	1430	1943	2162	2381	2703	2976	3516	3994	4469	0.0016 †
Gyeonggi	5040	6081	7158	9155	10,285	11,383	12,637	14,483	17,247	19,960	22,648	0.0028 †
Stomach cancer (C16)												
Seoul	35,321	26,280	29,893	33,537	36,202	38,635	40,572	42,553	44,862	46,937	49,125	-
Incheon	5471	6345	7271	8495	9408	10,247	11,074	12,014	12,954	13,863	14,587	0.0006 †
Gyeonggi	24,913	28,556	32,415	37,223	40,777	44,356	47,975	52,409	56,814	61,123	65,256	0.0007 †
Liver cancer (C22)												
Seoul	8707	6847	7863	9173	9803	10,314	10,784	11,072	11,748	12,255	12,867	-
Incheon	1235	1428	1618	2024	2253	2441	2649	2866	3125	3341	3593	0.0008 †
Gyeonggi	6109	6990	7923	9637	10472	11,234	12,172	13,405	14,643	15,847	17,166	0.001 †
Colorectal cancer (C18–C20)												
Seoul	29,616	23,809	27,430	31,386	34,184	36,679	38,701	40,832	43,364	45,464	47,776	
Incheon	4338	5286	6172	7330	8169	8939	9838	10,703	11,680	12,498	13,331	0.0007 †
Gyeonggi	20,114	24,185	27,658	32,356	35,947	39,365	42,932	47,007	51,212	55,489	59,600	0.0007 †
Breast cancer (C50)												
Seoul	27,934	25,289	28,356	31,351	33,986	36,830	39,545	42,405	46,195	49,868	53,704	-
Incheon	4655	5670	6366	7310	8138	8978	9902	10,969	12,141	13,291	14,539	0.0009 †
Gyeonggi	20,151	24,533	2,7629	31,708	35,299	39,232	43,240	48,512	54,751	60,794	67,010	0.0013 †
Cervical cancer (C53)												
Seoul	8887	7328	7779	8203	8497	8706	8925	9072	9367	9625	9806	-
Incheon	1717	1998	2130	2315	2447	2546	2671	2775	2960	3068	3174	0.1067
Gyeonggi	6872	7751	8204	8924	9454	9993	10,482	11,166	11,913	12,660	13,240	0.0689
Cancer of the esophagus (C15)												
Seoul	1131	777	901	1106	1223	1318	1416	1517	1735	1872	2041	-
Incheon	173	190	215	281	324	358	376	425	446	485	566	0.0009 †
Gyeonggi	720	770	878	1125	1240	1373	1531	1719	1970	2203	2430	0.0014 †
Gallbladder cancer (C23–C241)												
Seoul	893	702	855	1129	1332	1433	1559	1664	1985	2198	2516	-
Incheon	115	148	182	290	324	373	411	417	504	576	658	0.0013 †
Gyeonggi	558	689	851	1215	1411	1518	1681	2018	2371	2796	3120	0.0025 †
Pancreatic cancer (C25)												
Seoul	1724	1282	1365	1513	1621	1698	1771	1817	1912	1983	2074	-
Incheon	292	341	371	409	433	456	473	492	522	561	609	0.1118 †
Gyeonggi	1223	1344	1455	1637	1771	1846	2001	2181	2299	2438	2551	0.0519 †
Laryngeal cancer (C32)												
Seoul	3177	2609	3209	3869	4403	5012	5601	6229	6960	7800	8543	-
Incheon	545	646	786	962	1133	1285	1448	1642	1840	2100	2318	0.0009 †
Gyeonggi	2076	2663	3194	3896	4539	5293	6057	7091	8121	9300	10,500	0.0015 †
Small intestine cancer (C17)												
Seoul	1404	1207	1407	1590	1776	1938	2102	2290	2544	2735	2926	-
Incheon	222	307	344	410	455	506	561	640	703	787	844	0.0009 †
Gyeonggi	1050	1288	1457	1734	1913	2095	2341	2598	2983	3388	3668	0.0017 †
Skin cancer (C44)												
Seoul	3371	2899	3176	3469	3703	3961	4212	4393	4679	5014	5266	-
Incheon	542	670	734	836	904	982	1091	1161	1285	1355	1431	0.0006 †
Gyeonggi	2583	3100	3355	3775	4109	4468	4839	5306	5833	6312	6816	0.0009 †
Renal cancer (C64–C68)												
Seoul	9201	7833	9477	11,065	12,463	13,659	14,819	16,225	18,075	20,110	22,451	-
Incheon	976	1123	1375	1641	1913	2145	2436	2789	3261	3767	4328	0.0024 †
Gyeonggi	5354	6842	8174	9844	11,243	12,713	14,307	16,290	18,672	21,244	24,094	0.0016 †
Leukemia (C91–C95)												
Seoul	4410	3677	4286	4839	5360	5886	6335	6900	7590	8228	8926	-
Incheon	566	730	854	1042	1163	1325	1500	1651	1907	2110	2385	0.0011 †
Gyeonggi	2876	3619	4165	4867	5519	6137	6875	7784	8884	10,041	11,234	0.0017 †
Thyroid cancer (C73)												
Seoul	5917	4763	5368	6053	6532	6967	7050	7857	8449	9114	9805	-
Incheon	839	977	1124	1326	1493	1606	1775	1945	2168	2371	2600	0.001 †
Gyeonggi	3880	4567	5125	5911	6554	729	7961	8686	9674	10,642	11,656	0.0009 †

† *p* < 0.001.

## References

[1] Han Y.Q., Zhu T. (2015). Health effects of fine particles (PM 2.5) in ambient air. SCIENCE China Life Sci..

[2] Dae L.J. (2010). A Study on Indoor Air Quality in School. J. Korean Soc. Indoor Environ..

[3] Won W.S., Oh R., Lee W.J., Kim K.Y., Ku S.K., Su P.C., Yoon Y.J. (2020). Impact of Fine Particulate Matter on Visibility at Incheon International Airport, South Korea. Aerosol Air Qual. Res..

[4] Lee B.K., Kim Y.H., Ha J.Y., Lee D.S. (2005). Development of an Automated and Continuous Analysis System for PM 2.5 and Chemical Characterization of the PM 2.5 in the Atmosphere at Seoul. J. Korean Soc. Atmos. Environ..

[5] Shin M.-K., Lee C.-D., Ha H.-S., Choe C.-S., Kim Y.-H. (2007). The Influence of Meteorological Factors on PM10 Concentration in Incheon. J. Korean Soc. Atmos. Environ..

[6] Park E.-J., Kang M., You D.-E., Kim D.-S., Yu S.-D., Chung K.-H., Park K. (2005). Health Risk Assessment of Heavy Metals in Fine Particles Collected in Seoul Metropolitan Area. Environ. Anal. Health Toxicol..

[7] Yi O., Hong Y.-C., Kim H. (2010). Seasonal effect of PM 10 concentrations on mortality and morbidity in Seoul, Korea: A temperature-matched case-crossover analysis. Environ. Res..

[8] Zanobetti A., Schwartz J. (2005). The effect of particulate air pollution on emergency admissions for myocardial infarction: A multicity case-crossover analysis. Environ. Health Persp..

[9] Oh K.J., Kwak J., Jung D.Y., Son G.T. (1998). Statistical Analysis between Air Pollutants and Meteorological Factors in Pusan—Focusing at Kwanganli Area. J. Korean Soc. Environ. Eng..

[10] Lee J.T., Shin D.C., Chung Y. (1999). Air pollution and daily morality in Seoul and Ulsan, Korea. Environ. Health Perspect..

[11] Lee J.T., Kim H. (2001). Epidemiology Methods and Study Desings for Investigating Adverse Health Effects of Ambient Air Pollution. Korean J. Prev. Med..

[12] Moon S.W. (2016). Climate Change and Psychological Adaptation: Psychological Response, Adaptation, and Prevention. J. Korean Soc. Atmos. Environ..

[13] Kim Y.U., Lee H., Jang Y.J., Lee H.J. (2016). A Cluster Analysis on the Risk of Particulate Matter Focusing on Differences of Risk Perceptions and Risk-Related Behaviors based on Public Segmentation. J. Public Relat..

[14] Ham J.Y., Lee H.J., Cha J.W., Ryoo S.-B. (2017). Potential Source of PM10, PM2.5, and OC and EC in Seoul During Spring 2016. J. Atmos. Korean Meteor. Soc..

[15] Kim H.K., Jung K.M., Kim T.S. (2006). Characteristics of Seasonal Distributions of Fine Particles (PM 2.5) and Particle-Associated Polycyclic Aromatic Hydrocarbons inUrban, Metropolitan and Industrial Complex Sites. J. Environ. Toxicol.

[16] Park S.-M., Moon K.-J., Park J.-S., Kim H.-J., Ahn J.-Y., Kim J.-S. (2012). Chemical characteristics of ambient aerosol during Asian Dusts and high PM episodes at Seoul intensive monitoring site in 2009. J. Korean Soc. Atmos. Environ..

[17] Kang C.-M., Park S.-K., Sun W.Y., Kang B.-W., Lee H.-S. (2006). Respiratory health effects of fine particles (PM 25) in Seoul. J. Korean Soc. Atmos. Environ..

[18] LEE J.T., Kim H., Hong Y.C., Kwon H.J., Schwartz J., Christianie D.C. (2000). Air pollution and daily mortality in seven major cities of Korea, 1991–1997. Environ. Res..

[19] Lee H.S., Kang B.W. (2001). Chemical Characteristics of principal PM_2.5_ species in Chongju, Seoul Korea. Atmos. Environ..

[20] Jeon B.I. (2012). Meteorological Characteristics of the Wintertime High PM10 Concentration Episodes in Busan. J. Environ. Sci. Int..

